# To do or not to do – a survey study on factors associated with participating in the Danish screening program for colorectal cancer

**DOI:** 10.1186/s12913-020-06023-6

**Published:** 2021-01-07

**Authors:** Jesper Bo Nielsen, Gabriele Berg-Beckhoff, Anja Leppin

**Affiliations:** 1 Research Unit for General Practice, Department of Public Health, University of Southern Denmark, J.B.Winsløwsvej 9A, DK-5000 Odense, Denmark; 2 Research Unit for Health Promotion, Department of Public Health, University of Southern Denmark, Esbjerg, Denmark

**Keywords:** Colorectal cancer, Screening, Population, Survey, participation rates, Socio-demography, Barriers, Obesity, Risk aversity, Fecal immunochemical test (FIT)

## Abstract

**Background:**

Screening programs for colorectal cancer (CRC) exist in many countries, and with varying participation rates. The present study aimed at identifying socio-demographic factors for accepting a cost-free screening offer for CRC in Denmark, and to study if more people would accept the screening offer if the present fecal test was replaced by a blood test.

**Methods:**

We used a cross-sectional survey design based on a representative group of 6807 Danish citizens aged 50–80 years returning a fully answered web-based questionnaire with socio-demographic data added from national registries. Data were analyzed in STATA and based on bivariate analyses followed by regression models.

**Results:**

Danes in general have a high level of lifetime participation (+ 80%) in the national CRC screening program. The results of the stepwise logistic regression model to predict CRC screening participation demonstrated that female gender, higher age, higher income, and moderate alcohol intake were positively associated with screening participation, whereas a negative association was observed for higher educational attainment, obesity, being a smoker, and higher willingness to take health risks. Of the 1026 respondents not accepting the screening offer, 61% were willing to reconsider their initial negative response if the fecal sampling procedure were replaced by blood sampling.

**Conclusion:**

The CRC screening program intends to include the entire population within a certain at-risk age group. However, individual factors (e.g. sex, age obesity, smoking, risk aversity) appear to significantly affect willingness to participate in the screening program. From a preventive perspective, our findings indicate the need for a more targeted approach trying to reach these groups.

**Supplementary Information:**

The online version contains supplementary material available at 10.1186/s12913-020-06023-6.

## Background

Since 2014, Danish citizens have been invited to participate in screening for colorectal cancer (CRC) once they reach 50 years, and from 2018 this offer is repeated every second year until the age of 74. Participation is free of charge and the invitation for the Fecal Immunochemical Test (FIT) includes information on incidence of colon cancer and treatment options, an instruction (text and graphics) on sampling, all materials needed for the fecal test, and a prepaid return envelope. Those respondents in whose fecal samples the FIT detects blood (> 100 ng hemoglobin/mL [1]) are subsequently invited to a state-funded colonoscopy at their local hospital.

Thus, in contrast to many other countries where programs need to be paid out of pocket or where significant population subgroups may lack health insurance (for example Pakistan [2]), South Korea [3] or the US [4] there should not be financial barriers for accepting the screening offer in the Danish setting. However, despite the free offer, around 40% of Danes do not participate when they are initially invited [5]. There is hence a keen interest to identify factors associated with participation.

Female gender and older age have consistently been found to increase screening uptake in 12 countries [6]. Likewise, lifestyle behavior, health-related attitudes and cognitive styles seem to make a difference [7]. For health literacy, however, most studies failed to show an expected positive association with participation in screening for CRC [8, 9], but methodological differences across studies in the assessment of health literacy challenge inter-study comparisons [8].

Another factor which could act as a barrier for participation is the method of testing itself. When comparing the two feces-based FOBt and FIT methods, it turned out that uptake among non-responders almost doubled with shift from FOBT to FIT [10], with the latter not only being more specific but requiring only one instead of three feces samples and involving less dietary and medication restrictions prior to testing. It therefore seems relevant to examine if moving away from feces as testing material might potentially increase intention to participate in future colorectal screening.

## Aims

The research questions for the present study in a Danish setting were: (1) Are socio-demographic factors, health behaviors, subjective health status, health literacy and willingness to take health risks important factors for accepting the offer to be screened for CRC? (2) Would more people intend to accept the screening offer if the FIT was replaced by a blood test?

## Methods

### Sample and procedure

**Fig. 1:**
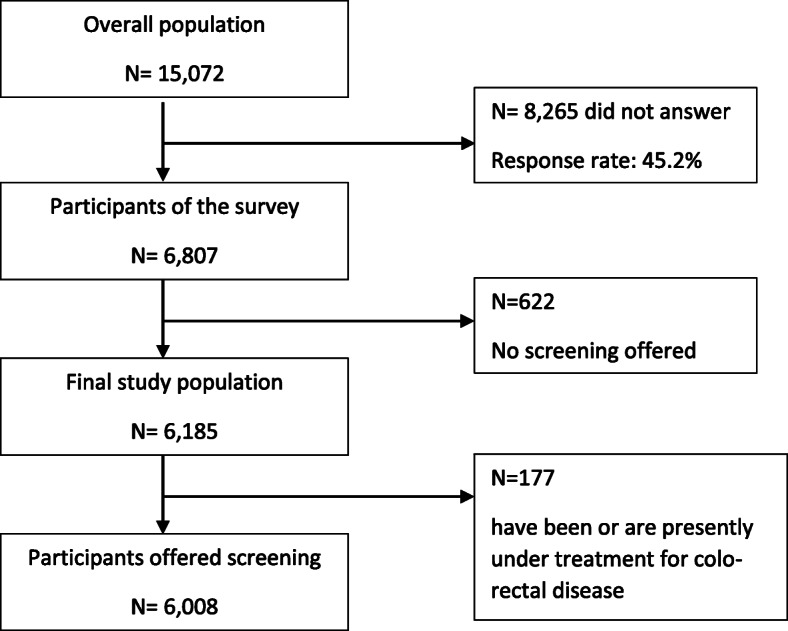
Flow chart population characteristic of the survey

We used a cross-sectional survey design based on a representative group of 15,072 Danish citizens aged 50–80 years. The national digital mailbox for official communication from governmental agencies to citizens was used in the present study. Data were collected in 2019 through a web-based standardized questionnaire (digital mail) administered by Statistics Denmark, and socio-demographic data including birthplace and residence were added from national registries. Two reminders were sent through digital mail. An English version of the Danish questions can be found as a supplementary file to this paper. Among the net sample, 6807 persons (45%) returned a fully completed questionnaire. Of these, 6185 had been offered a screening for CRC. Six hundred twenty-two had not yet received a screening offer and were therefore excluded. In the screened group 177 participants were excluded due to current treatment for colorectal disease (see Fig. 1). Besides being able to read and understand the Danish language, these were the only exclusion criteria. All analyses are based on 6008 participants.

According to the Act on a Biomedical Research Ethics Committee System in Denmark, the project was not a biomedical research project and did not need the ethic committee’s approval. Data include information that could potentially identify individuals, and the project is therefore registered at the University’s Research and Innovation Office, and data handling is in accordance with the General Data Protection Regulation (GDPR) from (EU) 2016/679.

### Variables

Main outcome was uptake of the screening offer (no/yes), which was assessed by self-report data from a standardized online questionnaire.

Register information included birthplace, place of residence, age, sex, highest educational attainment, and average individual income.

All health-related information was based on self-reported data. Self-assessed health was measured by a standard single item: *“How would you rate your current state of health?”* which was rated on a 5-point Likert scale: “nearly perfect”, “very good”, “good”, “poor”, or “very poor” and was afterwards dichotomized into “good” and” poor”. Body mass index (BMI) was calculated from self-reported weight (kg) and height (meters). It was categorized according to WHO criteria [11] into three levels: < 25, 25–29.99 (overweight), and ≥ 30 (obese). The propensity to take health risks was assessed by the question “How do you evaluate your willingness to take a risk related to your health situation?” Participants could answer on a scale from 0 (no risk willingness) to 10 (high risk willingness). Scores between zero to four were coded as “low”, five and six as “moderate” and seven points and more as “high willingness to take a risk”. Health literacy assessment was based on four Likert-scale questions considering health literacy in disease management about (1) finding information about diseases, (2) finding professional help when ill, (3) a good understanding when communicating with physicians and (4) understanding how to use medicine. Cronbach’s alpha for the 4-item health literacy scale was 0.83. The sum scores were dichotomized as “adequate” (< 8 points) or “non-adequate”. Smoking habits were assessed with a single item: *“Do you smoke?”*. The variable was coded with three levels: “current smoker”, “quit smoking”, and “never smoked.” Alcohol consumption was also asked for by a single item “How many units (equivalent to one glass of wine) of alcohol do you usually drink in a week?”, and answers were categorized into “none”, “1-14 units per week” and “more than 14 units per week” following the Danish recommendations [12]. For self-assessed healthy nutrition, participants were asked: “How do you evaluate your dietary habits?” Participants could answer either ‘very healthy’, ‘healthy’, ‘reasonably healthy’, unhealthy’, or ‘very unhealthy’. Answers were dichotomized into “healthy” and “unhealthy”. Physical activity was measured by: “On how many days of the past week did you engage in exercise for at least 30 minutes?” Responses ranged from 0 to 7 days. We used a cut-off of ≥5 days/week as criterion based on the physical activity guidelines for adults by the American College of Sports Medicine and the American Heart Association [13].

### Statistical analyses

All analyses were conducted in STATA version 16.0. Predictors for participation were initially tested on a bivariate level via chi-square-tests. Power calculation supported 80% power to find a significant (*p* < 0.05) OR of 1.7 or higher and would have allowed for an OR > 1.4 to become significant at a lower level of significance (*p* < 0.1) [14] . Stepwise logistic regression was used to test which of the variables independently affected screening participation. Additionally, a multinomial logistic regression was used on the offer to choose a blood test instead of the FIT (considering three answer options: “no”, “yes”, and “don’t know”). In both models we used a stepwise regression with a *p*-value of 0.1 for variable inclusion, which means that variables with a *p*-value above 0.1 were dropped from the final model. This is a common procedure in prediction analysis to identify the most important predictors [15]. Significance levels for testing individual factors were set at *p* = 0.05.

## Results

### Sample characteristics

Of the initial sample of 6807 respondents, 52.5% were females, which corresponds with the equivalent Danish population of the selected three age decades (Table 1). The mean age of respondents was slightly higher than in the respective Danish population segment, which was due to a slightly higher participation in the age group from 61 to 70 years (Table 1). Our sample was slightly better educated than the comparator, and a slightly higher percentage was still active on the labor market (Table 1). Birthplace and place of residence (geographical) were very close to the comparator (Table 1).

**Table 1:** Sample characteristics. Study sample (*n* = 6008), gross sample of 50–80 years olds (*n* = 6807) compared to the same-aged Danish population (*n* = 2.054.477)

	Study sample (%)	Gross sample (%)	DK population (%)
Sex			
Female	52.9	52.5	50.9
Age			
50–60	39.9	38.8	42.3
61–70	37.4	36.6	32.1
71–80	22.7	24.6	25.6
Education			
< 10 years	18.5	18.9	27.3
11–13 years	44.2	44.0	43.8
> 13 years	37.3	37.1	28.9
Work status			
Working		51.7	48.3
Not working		48.3	51.5
Personal income			
< 27.000 Euro		34.9	42.3
27.000–40.000 Euro		32.7	30.6
> 40.000 Euro		32.4	27.0
Birthplace			
Denmark		94.9	92.0
Residence in DK			
Capital (Copenhagen)		28.7	28.3
Zealand		16.1	16.3
Jutland + Funen		55.2	55.3

### Predictors for colorectal cancer screening

Among respondents, 82.6% had participated at least once in the Danish CRC-program. Those offered the CRC screening (*n* = 6008) due to having reached the age threshold were older, more often female (54.4%), less often had a higher education, and more often had high income (Table 2). Regarding the health situation of CRC participants, they were less often obese, had a good self-assessed health status, and less often described themselves as taking risks with their health compared to survey respondents who did not participate in CRC screening. No difference between participants and non-participants in CRC screening was seen in health literacy. There was, however, a generally very high prevalence of adequate health literacy among respondents (82.9%). Non-smokers as well as respondents who described themselves as eating healthily were more often seen among participants in CRC screening than smokers and those who reported less healthy eating habits. No difference was visible regarding levels of physical activity. In connection to alcohol consumption, total abstainers were found less often among participants in CRC screening, whereas respondents with an average weekly alcohol consumption of 1–14 units more often participated in the CRC screening (Table 2).

**Table 2:** Characteristics of the population who received an offer for CRC screening (*n* = 6008)

Characteristics	Participants		Non- participants		Total		
	n	%	N	%	N	%	***p***-value*
**Overall (row-%)**	**4971**	**82.62**	**1037**	**17.38**	**6008**	**100.00**	
**Sex**							
Female	2701	54.35	475	45.81	3176	52.86	< 0.0001
**Age groups**							
50–60	1916	38.54	483	46.58	2399	39.93	
61–70	1890	38.02	357	34.43	2247	37.40	
71–80	1165	23.44	197	19.00	1362	22.67	< 0.0001
**Education**^**a**^							
Basic school	919	18.49	192	18.51	1111	18.49	
High school	2195	44.16	436	42.04	2631	43.79	
Vocational education	276	5.55	37	3.57	313	5.21	
Medium education	1114	22.41	239	23.05	1353	22.52	
High education	467	9.39	133	12.83	600	9.99	0.001
**Average income per year (Euro)**							
< 20,000	294	5.91	90	8.68	384	6.39	
20,000 – 33,333	1508	30.34	353	34.04	1861	30.98	
33,334 – 46,666	1505	30.28	295	28.45	1800	29.96	
46,667 – 66,666	1158	23.30	209	20.15	1367	22.75	
> 66,666	506	10.18	90	8.68	596	9.92	< 0.0001
**Body mass index**							
< 25	2062	41.48	405	39.05	2467	41.06	
25–30 (overweight)	1933	38.89	373	35.97	2306	38.38	
> 30 (obese)	892	17.94	232	22.37	1124	18.71	0.001
Unknown^b^	84	1.69	27	2.60	111	1.85	
**Self-assessed health**							
Good health	4265	85.80	859	82.84	5124	85.29	0.014
**Health literacy score**							
Sufficient (< 8)	4106	83.15	839	81.46	4945	82.86	0.19
**Willingness to take health risks**							
Low	3772	75.99	719	69.33	4491	74.84	
Moderate	820	16.52	207	19.96	1027	17.11	
High	372	7.49	111	10.70	483	8.05	< 0.0001
**Smoking status**							
Never smoked	2986	60.06	528	50.92	3514	58.49	
Quit smoking	1342	27.00	277	26.71	1619	26.95	
Smoker	643	12.94	232	22.37	875	14.56	< 0.0001
**Self-assessed nutrition**							
Healthy and very healthy	2910	58.54	537	51.78	3447	57.37	< 0.0001
**Physical activity**							
5 times a week and more	718	14.44	140	13.50	858	14.28	0.43
**Alcohol consumption**							
None	1109	22.31	298	28.74	1407	23.42	
1 to 14 units per week	2462	49.53	442	42.62	2905	48.34	
> 14 units per week	1400	28.16	297	28.64	1697	28.25	< 0.0001

*Chi square test comparing people participating and not participating in screening

^a^ “medium education” includes tertiary and bachelor’s education, and “high education” includes master and PhD-educations

^b^ no information on weight or height available, this group was included in the analysis to omit selection bias due to missings

The results of the stepwise logistic regression model to predict CRC screening participation, overall and stratified for gender, are presented in Table 3. Female sex, higher age, higher income, and moderate alcohol intake were positively associated with screening participation, whereas a negative association was observed for higher educational attainment, obesity, being a smoker, and higher willingness to take health risks. When subdivided into educational attainment levels, the positive association between income and screening participation was evident in all individual educational groups (data not shown). In all income groups, the lowest participation rate was observed among respondents with the highest educational attainment (data not shown). The variables self-assessed health, health literacy, physical activity, healthy nutrition were dropped from the final model due to their minor contribution in explaining screening participation. Factors significantly associated with more participation in CRC screening in men were older age and higher income, while better self-reported nutrition was associated with less participation. In women, higher education and obesity were associated with less participation.

**Table 3:** Stepwise logistic regression model for factors associated with participation in CRC-screening (*n* = 6008) overall and stratified between males and females

Characteristics			Stratified			
	Overall		Male		Female	
	OR	95%- CI	OR	95%- CI	OR	95%- CI
**Sex**						
Female	**1.46**	**1.26–1.70**				
**Age groups**						
50–60	1	Ref	1	Ref		
61–70	**1.37**	**1.17–1.61**	**1.43**	**1.15–1.79**		
71–80	**1.53**	**1.26–1.86**	**1.87**	**1.42–2.45**		
**Education**^**a**^						
Basic school	1	Ref	1	Ref	1	Ref
High school	0.98	0.81–1.19	1.09	0.84–1.42	0.86	0.65–1.15
Vocational education	1.41	0.96–2.07	**2.06**	**1.22–3.48**	0.86	0.49–1.53
Medium education	**0.76**	**0.61–0.95**	0.93	0.66–1.29	**0.64**	**0.47–0.87**
High education	**0.56**	**0.42–0.72**	0.70	0.48–1.02	**0.43**	**0.29–0.64**
**Average income per year (Euro)**						
< 20,000	1	Ref	1	Ref		
20,000 – 33,333	1.13	0.86–1.48	1.34	0.91–1.97		
33,334 – 46,666	**1.47**	**1.11–1.93**	**1.80**	**1.22–2.65**		
46,667 – 66,666	**1.68**	**1.26–2.24**	**2.28**	**1.52–3.42**		
> 66,666	**1.87**	**1.33–2.64**	**2.10**	**1.32–3.38**		
**Body mass index**						
< 25	1	Ref			1	Ref
25–30 (overweight)	1.07	0.91–1.26			1.04	0.82–1.32
> 30 (obese)	**0.80**	**0.66–0.97**			**0.75**	**0.57–0.98**
**Willingness to take health risks**						
Low	1	Ref	1	Ref		
Moderate	0.84	0.71–1.01	0.80	0.63–1.02		
High	**0.76**	**0.60–0.96**	**0.73**	**0.54–0.99**		
**Smoking status**						
Never smoked	1	Ref	1	Ref	1	Ref
Quit smoking	0.91	0.77–1.07	0.99	0.79–1.24	0.82	0.65–1.04
Smoker	**0.57**	**0.47–0.68**	**0.65**	**0.50–0.84**	**0.49**	**0.37–0.63**
**Alcohol consumption**						
None	1	Ref	1	Ref	1	Ref
1 to 14 units per week	**1.40**	**1.19–1.66**	**1.51**	**1.17–1.94**	**1.43**	**1.15–1.78**
> 14 times per week	1.04	0.82–1.32	1.12	0.82–1.52	0.97	0.63–1.48
**Self-assessed nutrition**						
Healthy and very healthy			**0.81**	**0.66–0.98**		
Variables not included in model	Self-assessed health, health literacy, physical activity, healthy nutrition		Self-assessed health, health literacy, physical activity, BMI		Self-assessed health, health literacy, physical activity, healthy nutrition, self-assessed risky behaviour, age, income	

Bold OR are significant

^a^ “medium education” includes tertiary and bachelor’s education, and “high education” includes master and PhD-educations

### Screening method

Respondents who had not accepted the screening invitation for a FIT were asked whether they would consider screening for CRC if the initial test (FIT) were replaced by a blood test taken at one’s GP or at a laboratory. Of these 1026 respondents, 61% were now willing to reconsider their initial negative response, whereas 17% stood firm on saying “no” to the offer (Table 4). Facilitators for reconsidering the initial decision were higher education and a self-assessed healthy nutrition, whereas older age was a barrier to change the initial decision not to participate (Table 4).

**Table 4:** Stepwise multinomial logistic regression model for factors associated with hypothetical acceptance of a future CRC blood test in rejectors of the FIT test (*n* = 1026). Results are presented as ORs with 95% confidence intervals

	Would you consider screening for CRC next time you get the offer if the FIT test is replaced by a blood sample taken at your GP?		
	No	Yes	Don’t know
n (%)	170(16.6)	624 (60.8)	232 (22.6)
**Sex**			
Men	1		
Women		0.70 (0.49–1.01)	0.98 (0.64–1,48)
**Age groups**			
50–60	1		
61–70		**0.43 (0.29–0.66)**	0.83 (0.51–1.35)
71–80		**0.23 (0.15–0.37)**	**0.44 (0.26–0.76)**
**Education**^**a**^			
Basic school	1		
High school		**2.16 (1.35–3.46)**	**2.02 (1.18–3.47)**
Vocational education		0.99 (0.39–2.48)	0.88 (0.29–2.69)
Medium education		**1.95 (1.15–3.31)**	1.59 (0.86–2.94)
High education		**2.58 (1.35–4.94)**	2.02 (0.96–4.28)
**Body mass index**			
< 25	1		
Overweight (25–30)		1.27 (0.84–1.92)	0.85 (0.53–1.36)
Obese (> 30)		1.58 (0.95–2.41)	0.82 (0.46–1.49)
**Self-assessed nutrition**			
Healthy and very healthy		**1.65 (1.13–2.41)**	**1.90 (1.23–2.93)**
Variables not included in the model	Self-assessed health, self-assessed health risk behaviour, health literacy, income, smoking, alcohol consumption, physical activity		

Bold OR are significant

^a^ “medium education” includes tertiary and bachelor’s education, and “high education” includes master and PhD-educations

## Discussion

When societies offer screening programs for the entire population, they are trying to identify a relatively small number of individuals with early indication of disease, while the majority of the population will not have much benefit. It is therefore important to target people expected to be at increased risk and to identify determinants for accepting the screening offer.

For the overall study population, we identified a very high participation rate of 82,6%. The difference to the 65,3% participation rate reported by a Danish registry study for 2015/2016 [16] can be explained by the different criterion of “ever-use” (at least once) employed in the present study. In comparison to other countries [6, 7], Danes in general appear to have a high level of acceptance of the national colon cancer screening program. The overall high participation rate might be explained by a postal reminder which is sent every second year to non-participants. This might be an even more active alert for participation in CRC screening than an e-mail which has also been shown to be effective [17]. Caution must, however, be raised regarding such comparisons as different countries may have different organization and payment schemes for their screening programs.

In agreement with previous studies from Denmark and UK, we found that female gender was associated with increased participation in CRC screening [16, 18]. Women’s generally higher health-consciousness and preventive orientation might thus also manifest itself with respect to colon cancer screening. However, it also needs to be noted that a review by Wools et al. [7], including studies worldwide, found female gender rather to be a barrier than a facilitator, so findings about gender might be country-specific. The higher responsivity among older participants is in line with results from a 12-country study by Klabunde et al. [6] as well as by a review by Wools et al. [7] and might reflect a stronger awareness of older people about the fact that colon cancer risk increases with age and/or less restrictive time schedules among those who have left the labor market.

That high income was a positive predictor for screening participation in the entire study population as well as in individual educational subgroups is a finding consistent with the literature [7, 16, 19], but might nevertheless be considered surprising since participation in Denmark is free of charge. It is therefore likely that the influence of income is not a directly enabling one but might be mediated by differing subcultural norms, concerns and benefit expectations. Alternatively or additionally, groups with lower income might have other than direct financial opportunity costs. Thus, they might rather spend their time and energy on more imminent seeming problems or might not want to lose income if they work on an hourly basis or are self-employed.

An unexpected effect occurred for education. Commonly, a higher level of education has been identified as a facilitator for screening participation [7, 19], and this was also the case in a prior Danish study based on registry data [16]. In contrast to that, in our study we observed that among the total group as well as in all individual income groups, people with the longest education (more than appr. 13 years in school) participated to a lesser degree.

Reasons for this discrepancy remain speculative at this point. It is possible that the shift in the Danish program from FOBT to FIT in 2018 might have made a difference in terms of raising participation rates in the lower educated groups or else that critical media reporting in recent years [20] on a low predictive value of the test (too many false positives), unwarranted coloscopies, and a 1% risk of things going wrong during coloscopy, has specifically deterred higher educated population segments, who might reflect more on such information, not to say understand the numbers.

Further, a certain amount of selection bias may have played a role. Our study population showed some overrepresentation of the higher educated while the lower educated segment was underrepresented when compared to the reference population. In particular, immigrants have been largely excluded, since the questionnaire was in Danish only, and particularly non-Western immigrants are known to have lower average income and be less likely to attend screening programs (e.g. [16]).

It certainly appears particularly contradictory that the higher educational groups participated less while higher income was associated with more participation. When stratified for gender, the association related to educational attainment was driven mainly by the female segment of the study population, whereas the positive association with income was driven by the male participants only. Whether our observation is a spurious statistical finding, or whether there is a gender-specific difference in the influence of education and income on screening uptake will await further studies.

The large sample size allowed for subgroup analyses and two relevant barriers were identified. Among women, being obese (BMI > = 30 kg/m^2^) but not overweight (BMI 25–30 kg/m^2^) appeared to be a particular barrier for CRC screening. This is in line with studies from the UK or US, which also found that obese persons in general [20] or obese women in particular [21] are less likely to take up colon cancer screening.

Reasons for this association are most likely diverse. Obesity as well as non-participation in general health screenings might reflect some level of negligence towards health issues. Otherwise, the obese in the targeted age group are more likely to already suffer from other chronic diseases, such as diabetes and/or coronary artery/vascular diseases, which might make them less likely to participate in colon cancer screening [22] because they might focus on coping with their manifest disease instead of a new, “hypothetical” health risk. In any case, lower uptake rates in this particular group might be problematic, since obesity is a significant risk factor for colon cancer [23, 24].

A parallel effect was observed for smoking. Thus, those at higher risk for colon cancer, i.e. smokers, participated less than non-smokers. This finding agrees with results reported by comparable public cancer screening programs [25] and might be due to a tendency in smokers to have more pessimistic and avoidant beliefs about cancer [26].

Among men, having a moderate to high willingness to engage in or accept health risks was found to be a barrier for CRC screening. This finding is in line with prior studies indicating that a personal disposition like sensation-seeking is associated with more risky health behavior [27].

A recurrent issue when discussing uptake of screening offers has been whether people with inadequate health literacy would profit from written information to a similar degree as those with high health literacy. In our study, health literacy did not influence participation in the screening program. This contrasts with a previous review indicating that low health literacy is generally associated with poor cancer screening uptake [8]. The most positive explanation is that information provided in the Danish setting is sufficiently good to enable also people with more limited health literacy to read and understand the messages. However, the non-effect might also be due to a limited variance in health literacy as an adequate health literacy was observed among 83% of our sample, which is relatively high compared to samples from other countries [28].

Within the subgroup of those who had not participated, 61% expressed that if the FIT were replaced by a blood test, they would participate. The obvious explanation is that they prefer a blood test to a fecal test, which agrees with studies demonstrating that an unwillingness to deal with the collection of fecal matter is an important subjective reason not to participate [29]. The observed opinion shift could, however, also involve some degree of regret and/or some degree of social desirability by providing what is perceived of as the ‘right’ response to an (as yet) hypothetical decision situation.

A strength of the present study was the large sample (*n* > 6000) allowing for subgroup analyses. Further, a participation rate of 45% for this kind of internet distributed questionnaire study, while surely not optimal, is relatively high. Also, non-responder analyses revealed few differences between participants and non-participants suggesting no major selection bias. Moreover, the present study was part of a larger study on health-related issues, so participants were not biased by knowing that CRC screening was addressed when they decided to enter the study, nor did they know this while responding to the initial questions on individual attitudes and risk behavior. Another strength is that we included only participants who had already made an actual decision about screening participation, so our study is not based on hypothetical deliberations. As for limitations, we cannot exclude the existence of confounders, e.g. family history of cancer, which have not been controlled for in the present analyses. Further, the free access to screening will limit comparability to settings with out-of-pocket payment, but on the other hand also eliminate a potentially strong determinant from obscuring other potential influence factors. Eventually, we also cannot exclude any social desirability bias among the self-reported lifestyle factors included as well as answers towards the question about participation in screening which might have led to an overestimation of participation rates.

## Conclusion

The CRC screening program intends to include the entire population within a certain at-risk age group. The present study confirmed factors that have previously been described in the literature to be positively associated with participation in CRC screening. However, the present study also found that among women, obesity (BMI > = 30 kg/m^2^ but not overweight (BMI 25–30 kg/m^2^)) appeared to be a barrier for CRC screening. Among men, having a moderate to high willingness to engage in/accept health risks was found as a barrier for CRC screening. Thus, individual factors appear to significantly affect willingness to participate in the screening program.

Within the subgroup of those who had not participated in the screening program, 61% expressed that if they were offered a new chance where the FIT was replaced by a blood test, they would participate.

To the extent that the present findings can be reproduced, they appear interesting from a preventive perspective because they may lead to a more targeted approach trying to reach these groups. The observation also points to the more general experience from risk communication, that we must target and differentiate risk messages to reach all.

## Supplementary Information

**Additional file 1.**

## Data Availability

The datasets used and/or analyzed in the current study are available from the corresponding author on reasonable request.

## Abbreviations

BMI: Body Mass Index
CRC: Colorectal Cancer
FIT: Fecal Immunochemical Test
FOBT: Fecal Occult Blood Test
