# Supplementary material for: To do or not to do – a survey study on factors associated with participating in the Danish screening program for colorectal cancer
Source: BMC Health Serv Res. 2021 Jan 7;21:43. doi: 10.1186/s12913-020-06023-6 (PMC7792101; doi:10.1186/s12913-020-06023-6)
Supplement: Supplementary file 1 — Additional file 1. [file 12913_2020_6023_MOESM1_ESM.docx]

The present survey is based on survey-specific questions linked to sociodemographic questions from Danish National Registers by Statistics Denmark. The survey-specific questions included in the present paper was used in Danish in the Danish setting. The translated version is given below including the explanation as to grouping of data, which is also stated in the methods section of the paper.

Self-assessed health was measured by a standard single item: “How would you rate your current state of health?” which was rated on a 5-point Likert scale: “nearly perfect”, “very good”, “good”, “poor”, or “very poor” and afterwards dichotomized into “good” (“nearly perfect” + “very good” + “good”) and ”poor” (“poor” + “very poor”).

Willingness to take health risks was assessed by the question “How do you evaluate your willingness to take a risk related to your health situation?” Participants could answer on a scale from 0 (no risk willingness) to 10 (high risk willingness). Scores between zero to four were coded as “low”, five and six as “moderate” and seven points and more as “high willingness to take a risk”.

Health literacy assessment was based on four questions related to how easy/difficult the respondents experience (1) finding information about diseases that worries me, (2) finding out where I can get professional help when ill, (3) understanding what the doctor tells me, and (4) understanding instructions from doctor or pharmacist on how to use a medication. All questions were answered by four-point Likert-scales (“very easy”, “easy”, “difficult”, “very difficult”). The sum scores were dichotomized as “adequate” (<8 points) or “non-adequate”.

Smoking habits were assessed with a single item: “Do you smoke?” with three answering options: “yes”, “no, I have stopped smoking”, and “no I have never smoked.”

Alcohol consumption was assessed by a single item “How many units (equivalent to one glass of wine) of alcohol do you drink usually in a week?”, and answers were categorized into “none”, “1-14 units per week” and “more than 14 units per week”.

Self-assessed healthy nutrition was assessed by asking “How do you evaluate your dietary habits?” Participants could answer either “very healthy”, “healthy”, “reasonably healthy”, “unhealthy”, or “very unhealthy”’. Answers were dichotomized into “healthy” (“very healthy” + “healthy” + “reasonably healthy”) and “unhealthy” (“unhealthy” + “very unhealthy”).

Physical activity was measured by: “On how many days of the past week did you engage in exercise for at least 30 minutes?” Responses ranged from 0–7 days. We used a cut-off of ≥ 5 days/week as criterion for dichotomizing data based on the physical activity guidelines for adults by the American College of Sports Medicine and the American Heart Association.

Before answering questions specific to CRC, respondents were given basic information on incidence of colorectal cancer in Denmark, testing procedures including the offer of a subsequent coloscopy, frequencies of false positive (false alarm), and potential complications related to a subsequent coloscopy.

Participation in CRC screening was assessed with two items: “Have you within the most recent 5 years received an offer to be screened for colorectal cancer?”, which could answered by yes or no, followed by “Did you accept the offer to be screened for colorectal cancer?”. Participants could answer the latter question by “yes”, “no”, or “not relevant as I am/was already in a treatment or control process related to a colorectal disease”

Influence of testing method was assessed with a single item to those answering no to the question above: “Would you consider screening for CRC next time you get the offer if the fecal test sample is replaced by a blood sample taken at the GP/laboratory?”. Participants could answer “yes”, “no”, or “Don’t know”.
